# A Robust Method for Real Time Intraoperative 2D and Preoperative 3D X-Ray Image Registration Based on an Enhanced Swin Transformer Framework

**DOI:** 10.3390/bioengineering12020114

**Published:** 2025-01-26

**Authors:** Wentao Ye, Jianghong Wu, Wei Zhang, Liyang Sun, Xue Dong, Shuogui Xu

**Affiliations:** 1 China-UK Low Carbon College, Shanghai Jiao Tong University, Shanghai 200240, China; yewentao2000@sjtu.edu.cn (W.Y.); zhangv12@sjtu.edu.cn (W.Z.); liyang_sun@sjtu.edu.cn (L.S.); 2 Xinqiao Hospital, Chongqing 400037, China; 3 Shanghai Changhai Hospital, Shanghai 200433, China

**Keywords:** 2D–3D registration, X-ray, CT, Swin transformer, attention mechanisms, feature pyramid, image-guided surgery

## Abstract

In image-guided surgery (IGS) practice, combining intraoperative 2D X-ray images with preoperative 3D X-ray images from computed tomography (CT) enables the rapid and accurate localization of lesions, which allows for a more minimally invasive and efficient surgery, and also reduces the risk of secondary injuries to nerves and vessels. Conventional optimization-based methods for 2D X-ray and 3D CT matching are limited in speed and precision due to non-convex optimization spaces and a constrained searching range. Recently, deep learning (DL) approaches have demonstrated remarkable proficiency in solving complex nonlinear 2D–3D registration. In this paper, a fast and robust DL-based registration method is proposed that takes an intraoperative 2D X-ray image as input, compares it with the preoperative 3D CT, and outputs their relative pose in x, y, z and pitch, yaw, roll. The method employs a dual-channel Swin transformer feature extractor equipped with attention mechanisms and feature pyramid to facilitate the correlation between features of the 2D X-ray and anatomical pose of CT. Tests on three different regions of interest acquired from open-source datasets show that our method can achieve high pose estimation accuracy (mean rotation and translation error of 0.142° and 0.362 mm, respectively) in a short time (0.02 s). Robustness tests indicate that our proposed method can maintain zero registration failures across varying levels of noise. This generalizable learning-based 2D (X-ray) and 3D (CT) registration algorithm owns promising applications in surgical navigation, targeted radiotherapy, and other clinical operations, with substantial potential for enhancing the accuracy and efficiency of image-guided surgery.

## 1. Introduction

Image-guided surgery is a critical medical technique that employs medical images in different modalities to assist the surgeon in accurately visualizing and locating the area of operation, ensuring precision in surgical interventions [1]. Computed tomography (CT) images can offer three-dimensional insights into patient anatomy, rendering it exceptionally significant for preoperative surgical planning. In contrast, 2D (projected) X-ray images are frequently employed intraoperatively due to their lower radiation exposure, fast imaging, and the relative simplicity of acquisition, thus facilitating real-time confirmation and adjustment during surgical procedures. Since preoperative CT images offer extensive structural information, whereas intraoperative 2D X-ray images provide superior temporal immediacy, the matching (registration) of these two modalities can obtain better interventional guidance, which can help to reduce procedural invasiveness and increase the surgical precision [2]. For example, in external beam radiotherapy, CT-X-ray registration provides accurate inoperative patient positioning, which is crucial for delivering the precise dosage to the targeted area while minimizing exposure to critical healthy tissues [3]. Furthermore, in robot-assisted surgery, the registration results can be used to guide the robot to its trajectory planned from the preoperative CT images [4].

However, due to the different dimensions of CT and X-ray images, a direct comparison of their similarity is not feasible. Consequently, techniques such as dimensionality reduction or enhancement are commonly employed to adjust images from different modalities, facilitating accurate comparisons [5]. A widely recognized registration method uses a digitally reconstructed radiograph (DRR) to synthesize the simulated projected X-rays from preoperative CT volume, and comparing simulated X-rays with actual X-rays to estimate the alignment and posture [6]. In pose estimation, the optimization-based registration methods acquire the best spatial correspondence between two images by iteratively refining the pose guesses to minimize the similarity difference [7]. Powell’s method [8], the Nelder–Mead Algorithm [9], and the covariance matrix adaptation evolution strategy (CMA-ES) [10] are frequently employed to address complex nonlinear registration problems, particularly when gradients of the target function are difficult to access.

In recent years, researchers have incorporated additional prior information into the traditional algorithm to reduce the time required for registration. For example, Livyatan et al. [11] used volume gradient to eliminate outliers in X-ray fluoroscopic images, thereby achieving high registration accuracy. However, the fine gradient projection registration process based on edge pixels is time-consuming. Frysch et al. [12] employed Grangeat’s relation to integrate information both in 3D voxels and 2D projection and precomputed all time-intensive steps to accelerate the registration process. However, this method is susceptible to image truncation. Ban et al. [13] relied on the gradient direction of the 2D-weighted spatial histogram to extract statistical features, which expanded the convergence range when the organizational structure in the image’s foreground exceeds the image’s field of view, but this method has only been verified on a synthetic skull dataset with a small capture range. In general, traditional optimization-based methods can, to some extent, balance the trade-off between registration speed and accuracy by advanced parallel computing techniques and appropriate prior information. However, as the capture range in registration expands, the exploration space for optimization increases correspondingly, necessitating a greater number of optimization iterations to identify the optimal solution within more complex nonlinear search spaces. Consequently, both the accuracy and speed of optimization-based methods may be adversely affected.

The recent years have witnessed significant advancements in the accuracy and efficiency of medical image registration, driven by the integration of deep learning frameworks [14]. Deep learning models are trained to learn mappings from input images to optimal registration transformations. Once trained, these models can swiftly predict registered pose according to the input X-ray image without the time-consuming iterative processes. Hou et al. [15] trained a convolutional neural network (CNN) to directly learn the mapping between 2D CT slices and their 3D orientations. Miao et al. [16] hierarchically trained three levels of CNN models to regress pose residuals by comparing DRR with X-ray images. These methods focus on aligning a small area of interest, such as an implant, but their effectiveness in aligning larger areas of interest is somewhat limited due to their inadequate ability in capturing global semantic information. Liao et al. [17] introduced the point-of-interest network for tracking and triangulation which directly measured 3D deviations by extracting the point-to-point correspondence between multi-view CT images and X-ray images. However, this heavily relies on the precise annotation of the interest points across different dimensions during training, imposing stringent needs on the labeling of the training data. Learning-based registration methods have substantial potential because they can transfer the cumbersome computational demands from the intraoperative stage to the preoperative stage, making real-time cross-dimensional X-ray registration feasible. However, these methods often suffer from lower registration accuracy, exceeding a mean translation error of 5 mm within a 60 mm field of view, and a reliance on prior point-to-point correspondences. Therefore, it is necessary to develop a real-time robust registration method that not only achieves high registration accuracy and speed but also leverages data-driven approaches to ease the dependence on preoperative annotated points.

Recent advances in transformers have demonstrated their suitability for handling precise computer vision tasks within a large-scale training dataset [18]. The vision transformer (ViT) [19], which treats image patches as sequential data, enables global context modeling through self-attention. This global context capture has shown promise in complex tasks like image classification and registration, but its quadratic computational complexity makes it challenging for high-resolution images [20]. Swin transformer [21] is designed to address the limitations of ViT by introducing a hierarchical architecture and a shifted window mechanism, allowing multi-scale learning and information exchange between neighboring patches, thus reducing computational costs while maintaining effective local and global context modeling. Owning the capability to handle large-scale datasets and capture intricate details across multiple resolutions, Swin transformer demonstrates significant potential for medical vision tasks including medical image analysis [22] and segmentation [23].

In this paper, we introduce a dual-channel X-ray pose estimation Swin transformer (XPE-ST), an innovative learning-based approach specifically designed to address the current challenges in robust CT-X-ray registration tasks. To prepare the dataset, we generated 2,250,000 simulated X-ray images from their corresponding preoperative CT voxels using digitally reconstructed radiography (DRR) to form the training datasets. To design the registration method, X-ray pose estimation was formulated as a six-degrees-of-freedom (6-DOF: roll, pitch, yaw, dx, dy, dz) regression task, where the network was trained to learn the mapping between the extracted features and the corresponding pose parameters. To further enhance feature representation and maintain semantic consistency, channel attention mechanisms and a feature pyramid network (FPN) were integrated into the backbone of Swin transformer.

This article is structured as follows; Section 2 describes in detail the background and methodology of our registration farmwork, Section 3 shows the implementation details of our experiments, Section 4 analyzes and discusses the results of our experiments, and Section 5 gives our conclusions.

## 2. Methods

### 2.1. The 2D–3D X-Ray Image Registration

In the three regions of interest (head, chest, and pelvis) examined in this study, bones are strongly connected by ligaments and can thus be treated as rigid. Furthermore, since CT scans provide voxel-based data in 3D space and X-ray images capture pixel-based 2D projection, the registration from CT to X-ray in our work can be modeled as a rigid 2D–3D registration problem.

#### 2.1.1. Rigid Transformation

Rigid transformations in 3D space can be defined by six-degrees-of-freedom: three translation parameters $\left(dx,\;dy,\;dz\right),$ and three rotation parameters (α, β, θ). The translation parameters correspond to shifts along the x, y, and z axes of the Cartesian coordinate system and the rotation parameters represent angular changes around these axes, i.e., pitch, yaw, and roll. The above six parameters can be described by a matrix  T∈SE(3), as shown in Equation (1).

(1) $$T=\left[\begin{array}{cccc} cos\left(\beta \right)cos\left(\theta \right) & sin\left(\alpha \right)sin\left(\beta \right)cos\left(\theta \right)-sin\left(\theta \right)cos\left(\alpha \right) & sin\left(\alpha \right)sin\left(\theta \right)+sin\left(\beta \right)cos\left(\alpha \right)cos\left(\theta \right) & dx\\ sin\left(\theta \right)cos\left(\beta \right) & sin\left(\alpha \right)sin\left(\beta \right)sin\left(\theta \right)+cos\left(\alpha \right)cos\left(\theta \right) & -sin\left(\alpha \right)cos\left(\theta \right)+sin\left(\beta \right)sin\left(\theta \right)cos\left(\alpha \right) & dy\\ -sin\left(\beta \right) & sin\left(\alpha \right)cos\left(\beta \right) & cos\left(\alpha \right)cos\left(\beta \right) & dz\\ 0 & 0 & 0 & 1 \end{array}\right].$$

The core of the 2D–3D X-ray image registration is to estimate the transform matrix T so that it optimizes the similarity between images before and after transformation, which can be modeled as Equation (2), as follows:

(2) $$\hat{T}=arg\underset{T}{min}S\left(I_{\mathrm{target}},T\cdot I_{\mathrm{source}}\right),$$

where S represents the similarity difference between the target image $I_{\mathrm{target}}$ and the transformed source image $T\cdot I_{\mathrm{source}}$ and $\hat{T}$ is the optimal transform matrix estimated to minimum the similarity differences.

In our work, $I_{\mathrm{source}}$ represents a digitally reconstructed X-ray radiographic image (DRR) simulated from the 3D-CT volume and $I_{\mathrm{target}}$ represents the input 2D-X-ray image to be registered. Additionally, we have developed a coordinate system for the 2D–3D registration task to parameterize T in Equation (2).

#### 2.1.2. Digitally Reconstructed Radiograph (DRR)

The dimension discrepancy between preoperative CT data and intraoperative X-ray data poses a challenge for direct registration. As such, digitally reconstructed radiograph (DRR) is employed as a dimensionality reduction method to transform different modalities into a comparable dimensional space by simulating the X-ray projection process with CT volumes [5]. In the DRR process, assume that the X-ray follows a logarithmic response characteristic with no beam divergence. Such an assumption is valid and feasible, because the influence caused by beam divergence can be effectively modeled by various noise generation algorithm in the following procedure. As such, the entire imaging process can be modeled by Equation (3) [24], as follows:

(3) $$I\left(p\right)=\int \mu \left[L\left(p,r\right)\right]dr,$$

where I(p) represents the pixel intensity at a certain point p on the X-ray image; $L\left(p,r\right)$ denotes the path of an X-ray beam from the ray source r to point p; and μ(⋅) is the linear attenuation coefficient. Assume that J(x,y,z) is the linear attenuation field of the target object after X-ray transmission and $T_{Xray}^{CT}$ maps the transformation from the CT volume to the X-ray detector. Accordingly, the linear attenuation coefficient of p can be expressed as $\mu (p)=J[{T_{CT}^{X\text{-}ray}}^{-1}\cdot p]$. Substituting the coefficient into Equation (3), we can formulate Equation (4).

(4) $$I\left(p\right)=\int J\left[{T_{CT}^{X\text{-}ray}}^{-1}\cdot L\left(p,r\right)\right]dr,$$

**Figure 1:**
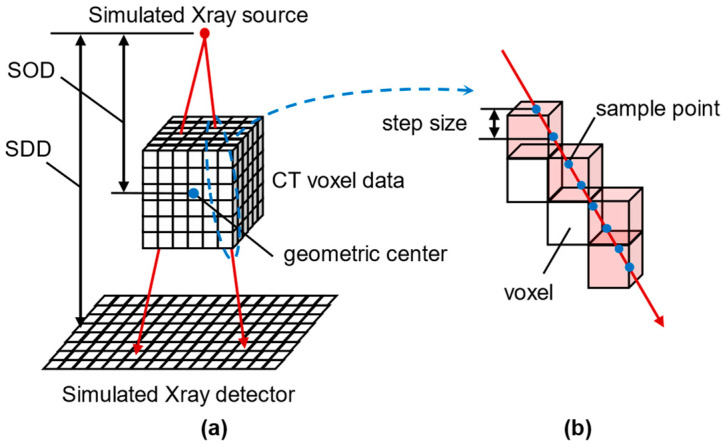
Digitally reconstructed radiograph (DRR) generation process: (**a**) the process of simulating projection and (**b**) the numerical line integration of each simulated X-ray beam passing through voxels.

Figure 1 presents a schematic diagram illustrating the method used to calculate the pixel intensity of each simulated X-ray image in DRR. The grayscale data in each CT voxel can be linearly converted to Hounsfield units [25], and as such, the linear attenuation field $J\left(x,y,z\right)$ can be obtained from the grayscale distribution in voxel. $T_{Xray}^{CT}$ can be calculated from the source-to-object distance (SOD) and source-to-detector distance (SDD) shown in Figure 1a, which can be obtained from the internal parameters. Given the X-ray source point and a certain pixel on the detector, the line $L\left(p,r\right)$ connecting these two points and intersecting the CT voxels can be readily determined. In summary, since all parameters in Equation (4) are known during the DRR process, the numerical line integration method can be employed (shown in Figure 1b) alongside the GPU-accelerated image-rendering technology developed by Kruger et al. [26], to efficiently generate simulated X-ray images.

#### 2.1.3. Coordinate Establishment

**Figure 2:**
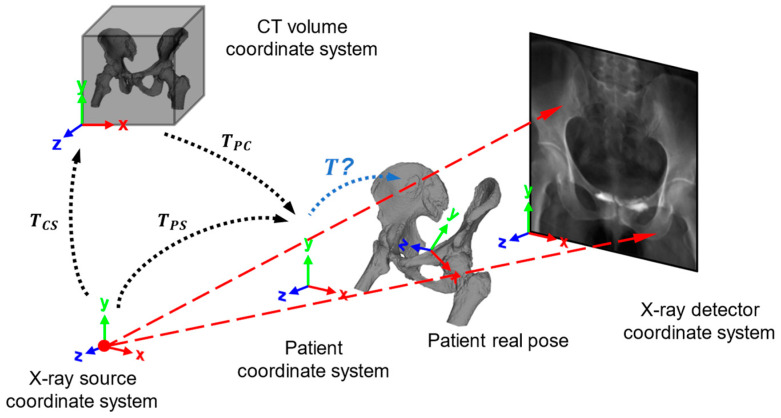
Coordinate establishment for CT-X-ray registration system, where T is the transform matrix to be estimated in $SE\left(3\right)$.

The relationships between the different coordinate system relevant to this study are illustrated in Figure 2.

The X-ray source and detector coordinate systems share the same orientation, whose xy axis is parallel to the detector’s xy axis, and the z axis points to the X-ray emission source. A customized patient coordinate system was defined between the source and the detector, aligned with the X-ray source and detector coordinate systems. The origin of patient coordinate system is placed along the z axis where z=SOD=0.5∗SDD. This design establishes a reference point for patient displacement, facilitating a more accurate description of the patient’s movement along the z-axis [27]. As such, the transform matrix T in Figure 2, which remains to be estimated, can be modeled as the pose transformation between the patient coordinate system and the actual patient’s position.

To incorporate the transformation T into DRR simulation process, it is essential to establish a relationship between the CT voxel and the patient coordinate system. Thus, the geometric center of the CT voxel is positioned at the origin of the patient coordinate system. A transform matrix $T_{PC}$, which can be obtained from the CT geometric parameters through Equation (5), is introduced to transform the CT volume coordinate system to the patient coordinate system, as follows:

(5) $$T_{PC}=\left[\begin{array}{cc} I & \begin{array}{c} -(a+\frac{L}{2})\\ -(b+\frac{W}{2})\\ -(c+\frac{H}{2}) \end{array} \end{array}\right],$$

where a,b and c refer to the origin position or offset in the CT volume coordinate system and L, W and H refer to the length, width and height of the CT volume.

Moreover, $T_{PS}$ is introduced as an intermediate transform matrix that model the translation from Xray source to patient coordinate origin, which is shown in Equation (6), as follows:

(6) $$T_{PS}=\left[\begin{array}{cc} R_{i} & \begin{array}{c} 0\\ 0\\ -\mathrm{SOD} \end{array} \end{array}\right],$$

where $R_{i}$ is the 3×3 extrinsic rotation matrix related to the primary and secondary angles in an X-ray shooting device like C-arm [28]. In our single-view X-ray projection setup, these angles are set to zero so $R_{i}=I$. Combining Equations (5) and (6), we can construct the relationship between the X-ray source and the CT voxel data for DRR simulation, which is shown in Equation (7).

(7) $$T_{CS}=T_{PC}^{-1}\cdot T_{PS},$$

#### 2.1.4. Problem Formulation

According to Section 2.1.3, all transformations involved in the DRR system are ultimately unified into our customized patient coordinate system. As such, the pose of the CT within the patient coordinate system, i.e., transform matrix T, can be adjusted to simulate the different X-ray images projected on different patient pose. If the registration and simulation process is entirely successful, the real X-ray image will perfectly align with the simulated X-ray image, and the transformation matrix applied during the simulation will precisely correspond to the actual transformation matrix that requires estimation.

It is evident that the CT-X-ray registration problem can be formulated as a regression task, where the goal is to find the six-degrees-of-freedom rigid pose parameters that best align the input X-ray image. These parameters are used to construct a transform matrix T, which is applied during the simulated X-ray projection process to maximize the similarity between the simulated X-ray image and input real X-ray image. Meanwhile, the estimated pose can be compared against the ground truth pose to evaluate the accuracy of the registration.

### 2.2. Registration System Framework

**Figure 3:**
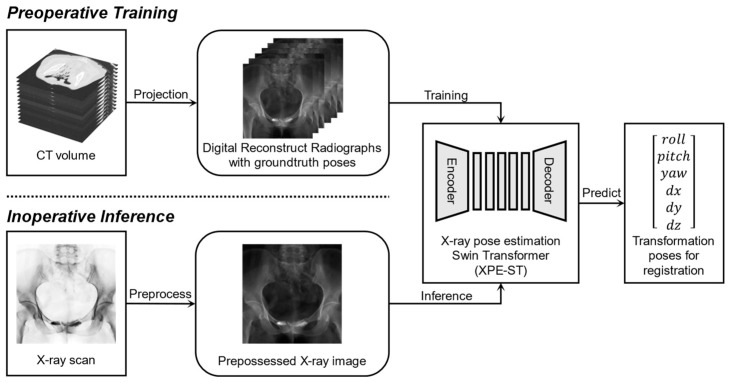
The structure of the registration framework that integrates preoperative training and intraoperative interference.

The proposed learning-based CT-X-ray registration system is illustrated in Figure 3. During the preoperative phase, CT volumes are used to generate a database of digitally reconstructed radiographs (DRRs) through Siddon’s [29] ray-casting method. This DRR database is primarily utilized for network training, with the projection parameters of each DRR serving as supervisory ground truth labels. During the intraoperative phase, the real X-ray image undergoes preprocessing (inversion, down-sampling, etc.), after which it is fed into the pre-trained X-ray pose estimation Swin transformer (XPE-ST) to directly predict the relative pose of the preoperative CT. Since this approach only requires loading the pre-trained model without any iterative calculations during the operation, the registration time can be reduced to under 0.5 s, meeting real-time requirements.

#### 2.2.1. XPE-ST Overview

**Figure 4:**
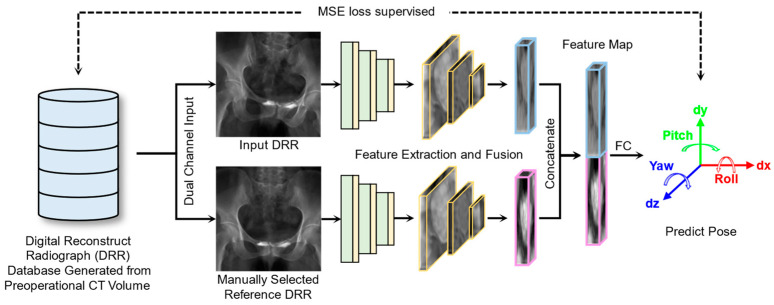
The architecture of X-ray pose estimation Swin transformer (XPE-ST).

Figure 4 shows the specific structure of XPE-ST. XPE-ST leveraged an advanced dual-channel Swin transformer backbone to effectively capture both local and global features of medical images. Furthermore, we introduced a feature fusion module for image registration, which incorporated both a channel attention mechanism and a feature pyramid network. The feature maps generated by feature extraction and fusion encoder were fully connected to produce a six-degrees-of-freedom (6-DOF) pose prediction, yielding high-precision registration results without reliance on any additional annotation points. The synergistic combination of customized modules aims to enhance the model’s capacity for robust image registration as well as improve the overall registration accuracy.

Since the network training utilized DRR images with a pre-defined ground truth pose ($Pose_{groundtruth}$), supervised training can be conducted as a data-driven method to progressively minimize the mean squared error (MSE) loss shown in Equation (8), thereby achieving optimal alignment between the X-ray image features and the registration posture.

(8) $$MSE=\frac{1}{n}\sum_{i=1}^{n}{\left(Pose_{groundtruth}-Pose_{predict}\right)}^{2},$$

#### 2.2.2. Dual Channel Image Input

During training and inference, the estimated image and a reference DRR image are fed into the network through two separate channels. This design ensures that the network can directly learn the feature and pose difference between input image pairs without explicitly modeling the relationship between the image and its pose, which is particularly well-suited for tasks requiring precise registration. In the selection of reference images, a DRR image with prominent features can be manually chosen as the reference. For example, in the case of the pelvis, such features may include the left and right hemipelvis, the upper and lower sacrum, and the vertebrae.

#### 2.2.3. Feature Extraction and Fusion Encoder

**Figure 5:**
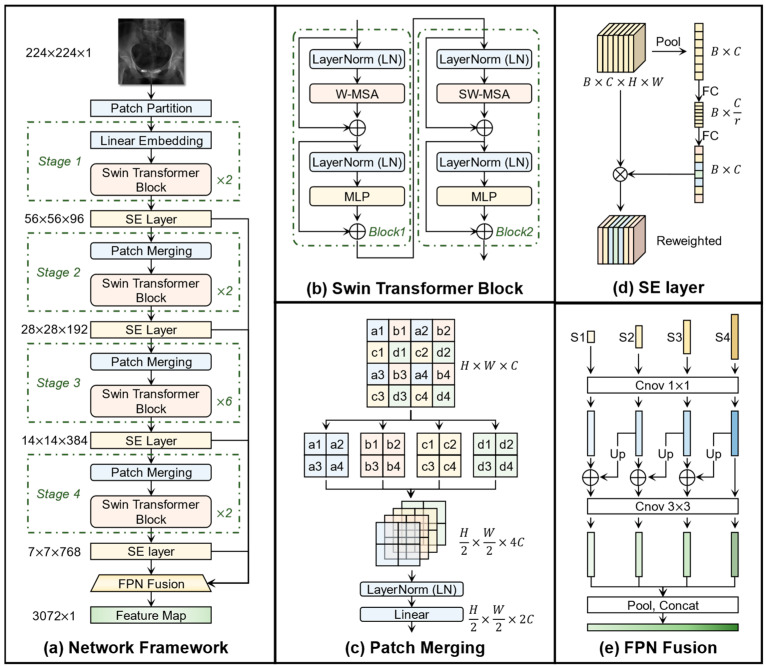
Detailed framework of the feature extraction and fusion encoder: (**a**) Whole framework with a Swin transformer backbone; (**b**) process in a Swin transformer block; (**c**) process of patch merging in a Swin transformer stage; (**d**) process of feature map reweighting based on the SE attention mechanism; (**e**) process of multi-layer feature fusion based on the feature pyramid network.

Figure 5 illustrates the detailed structure of each module within the feature extraction and fusion encoder. In a specific feature extraction channel, a single-channel grayscale image with a resolution of 224 × 224 serves as the input, which undergoes feature extraction, feature reweighting, and cross-stage feature fusion. The processed features are finally flattened and output as a 3072 × 1 feature map used for further parameter regression.

1. Feature extraction based on Swin transformer backbone

In Figure 5a, the input grayscale image is divided into patches within the patch partition process, where each group of 4 × 4 adjacent pixels form a patch, down-sampling the input image into the spatial dimensions of 56 × 56. This tokenization process makes the task well-suited for transformer architectures. Subsequently, four stages, which contain multiple Swin transformer blocks to refine the hierarchical representation, are designed to generate feature maps in varying sizes. In stage 1, a linear embedding layer is employed to expand the channel size into 96. In stages 2–4, a patch merging layer is used for down-sampling, to construct feature maps at different resolutions.

The structure of two adjacent Swin transformer blocks is shown in Figure 5b. Two different attention mechanisms, i.e., window-based multi-head self-attention (W-MSA) and shifted window-based multi-head self-attention (SW-MSA) [21], are sequentially employed across two consecutive Swin transformer blocks. In W-MSA, the input feature map is divided into fixed-size non-overlapping windows (7 × 7), and self-attention operations are performed independently within each window. The computational complexity of W-MSA is expressed in Equation (9), where h and w represent the height and width of the feature map, C denotes the depth of the feature map, and M is the size of each window. In contrast, the computational complexity of global multi-head self-attention (MSA) is shown in Equation (10). When the window size is appropriately selected, $M^{2}\ll hw$, leading to a significant reduction in computational complexity.

(9) $$O\left(MSA\right)=4hwC^{2}+2{\left(hw\right)}^{2}C,$$

(10) $$O\left(W-MSA\right)=4hwC^{2}+2M^{2}hwC,$$

However, W-MSA lacks a direct interaction between windows, which restricts cross-window information flow. To address this limitation, SW-MSA introduces a window-shift strategy to facilitate information transfer between windows. Specifically, SW-MSA shifts the window position by half the window size (i.e., by 3 or 4 pixels) in the adjacent layer, allowing previously independent windows to overlap and interact, thereby enhancing the model’s ability to capture global context and improving its feature representation capability. This sequential W-MSA-before-SW-MSA framework extends the contextual range and strengthens the correlation between features while maintaining computational efficiency, which explains why Swin transformer blocks should be equipped in pairs within each stage.

The feature map undergoes a patch merging process between two stages to facilitate the capture of multi-scale features. Patch merging combines neighboring patches into larger ones in the deeper layers, progressively reducing the spatial resolution while increasing the number of feature channels, akin to the pooling operation in convolutional neural networks, allowing the model to capture higher-level features at coarser scales. In Figure 5c, assuming the input feature map has the dimensions H×W×C, the process starts by dividing the feature map into patches of 2 × 2 pixels. Each pixel from the same position across the patches (indicated by the same color in the figure) is grouped together, resulting in four smaller feature maps of dimensions $\frac{H}{2}\times \frac{W}{2}$. These four feature maps are then concatenated along the depth dimension, increasing the number of channels from C to 4C. Finally, a normalization layer and a linear transformation are applied to reduce the channel depth from 4C to 2C to achieve the balance between feature dimensionality and computational efficiency.

In summary, the Swin transformer backbone employs two attention mechanisms, W-MSA and SW-MSA, in succession to simultaneously model the local and global features, effectively capturing crucial patterns. Additionally, patch merging enables multi-level feature extraction by creating feature maps with varying dimensions, facilitating the capture of information at different scales.

2. Feature map reweighting based on Squeeze-and-Excitation (SE) attention mechanism

The Swin transformer exhibits substantial depth, enabling channels across different layers to extract rich information. To weigh the importance of features in different channel, we introduced the squeeze-and-excitation (SE) channel attention mechanism [30] shown in Figure 5d. The SE block is designed to improve the representational power of a network by explicitly modeling the interdependencies between different channels of features.

After passing through a Swin transformer stage, the feature map with a batch-size B, channels C, width W, and height H can be expressed in the form of $\left(B,\;C,\;W,\;H\right)$. The feature map is then compressed to a (B, C) dimension feature vector group through a global pooling layer, which is called ‘squeeze’. In this squeeze step, the global information is embedded into a channel descriptor by aggregating feature maps across their spatial dimensions, which results in a descriptor that captures the global distribution of the feature responses.

A fully connected layer with a ReLU activation function is then applied to reduce the channels of squeezed feature vector group to C/r, where r is the manually defined hyperparameter reduction ratio. The reduction operation creates a bottleneck that forces the network to learn more compact and informative channel-wise dependencies, which helps in focusing on the most critical features and discarding less relevant information, leading to better generalization and performance. The second fully connected layer with a Sigmoid activation function is used to restore the channel dimensions back to C, which ensures each channel with a unique weight in the range [0,1]. The process involving two fully connected layers and two activation functions is called ’excitation’. During this excitation step, the channel descriptor passes through a simple gating mechanism, primarily designed using fully connected layers, to produce a set of modulation weights.

The weight vector obtained after the squeeze–excitation process is finally dimensioned and multiplied with the original feature map to obtain the reweighted feature map, thereby boosts the model’s ability to focus on the most relevant features after a Swin transformer stage.

3. Multi-layer feature fusion based on feature pyramid

Traditional regression networks typically utilize features from the last layer for subsequent tasks through fully connected layers. In contrast, to leverage both shallow and deep features throughout the whole network, we introduced the feature pyramid network (FPN) [31] to fuse features across different stages in the Swin transformer, as illustrated in Figure 5e.

Specifically, in FPN, each feature map reweighted by the SE attention mechanism is processed through a 1 × 1 convolutional layer to unify the channel dimensions. Following this unification, the lower resolution feature maps are up-sampled and integrated with the higher resolution maps from the preceding stage. This feature fusion process is bottom-up; the feature map from stage 4 (S4) is up-sampled and combined with the feature map from stage 3 (S3). Meanwhile, such upward integration is also carried out between S3 and stage 2 (S2) and between S2 and stage 1 (S1). Global pooling is then applied to each fused feature map, resulting in a 768 × 1 channel feature map for each layer. These feature maps are concatenated to form a comprehensive 3072 × 1 feature map, which serves as the output of the entire feature extraction and fusion encoder. As such, the output of each channel is a feature map that has been reweighted by channel attention and fused with multi-stage features. This feature map encapsulates multi-scale information from all stages of the Swin transformer backbone, allowing the network to incorporate both fine-grained details and high-level semantic information.

## 3. Experiments

### 3.1. Dataset Preparation

#### 3.1.1. CT Voxel Data Representing Three Distinct Anatomical Regions

To evaluate the generalizability of the proposed method, three distinct anatomical regions (head, chest, and pelvis) were selected, and corresponding open-source CT voxel datasets were acquired. The head CT scan was obtained from the HaN-Seg dataset [32], which comprises high quality medical images of 56 patients who underwent both CT and MR imaging. The chest CT scan was gathered from the BIMCV-COVID19+ dataset [33], which includes a collection of 1380 chest X-ray images and 163 CT images from COVID-19+ patients. The pelvis CT scan was acquired from CTPelvic1K dataset [34], consisting of 1184 high-resolution pelvic CT volumes.

In our experiments, a representative CT scan was selected from each region of interest to generate DRR images for training and testing. The geometric parameters of the selected CT images for each region are listed in Table 1. Notably, all raw data employed in this study are open-source and have been utilized in compliance with their respective license agreements. These datasets have been fully de-identified, ensuring the exclusion of any personally identifiable information. We extend our sincere appreciation to the data providers for their valuable contributions, which have been instrumental in enabling the present study.

#### 3.1.2. Training and Testing Data Preparation Through DRR

In the training phase, the specific CT voxel was positioned in various poses to simulate different X-ray projections, thereby generating DRR images in different orientations. These images, along with their corresponding projection pose parameters as supervising labels, collectively formed the training dataset for supervised learning. Based on the specific characteristics of different regions of interest, the capture range of the parameters (dx, dy, dz, roll, pitch, and yaw) for composing the projection pose was defined as detailed in Table 2. For the translation parameters, since the origin of the patient coordinate system is assumed along the z-axis, greater variability was expected along the z-axis. Therefore, the capture range for the translation parameter dz was set to be relatively wider to accommodate these variations. For the rotation parameters, since the in-plane rotation (yaw) around the z-axis is typically well-controlled in clinical practice, the capture range for this parameter was set to be relatively narrow.

During the image generation process, each pose parameter follows a normal distribution within the specified capture range. The number of training images to be generated was determined according to the width of the capture range, as also specified in Table 2. In the testing phase, we randomly generated 10,000 simulated X-ray images with varying poses within the capture range. These simulated X-ray images, not included in the training dataset, served as the input to the pre-trained model for modeling real X-ray inputs during testing. The accuracy of pose–parameter-regression was evaluated by comparing the differences between the predicted poses and the generated poses.

### 3.2. Implementation Details

To better simulate the actual X-ray imaging conditions, our DRR simulation parameters were aligned with the Siemens CIOS Fusion mobile C-arm. The simulated X-ray images had a resolution of 1536 × 1536 pixels with a pixel spacing of 0.194 mm, and a source-to-detector distance (SDD) of 1020 mm. The generated X-ray images were single-channel grayscale images, which were down-sampled to 224 × 224 pixels to meet the input requirements of the model.

During training, batch size was set to 128 with a 0.2 neuron dropout rate. The initial learning rate was set at 10^−5^. Adam [35] optimizer was used to minimize the loss during training. In our work, we used a NVIDIA Tesla V100 GPU with 30 GB VRAM for model training and used a NVIDIA 2080Ti GPU with 10 GB VRAM for model testing and regression performance assessment.

### 3.3. Comparative Experiments

**Figure 6:**
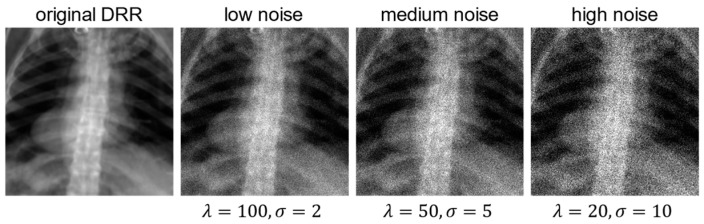
Comparison of the effects of different Poisson–Gaussian noise levels (low, medium, high) added to a DRR image, where λ represents the rate parameter of Poisson noise and σ denotes the standard deviation of Gaussian noise.

Multiple comparative experiments were conducted to assess the advantages of our registration method in terms of accuracy, robustness, and speed. We compared our proposed method (XPE-ST) with two traditional optimization-based approaches (Powell [8] and CMA-ES [10]) and two learning-based methods (parametric regression networks using ResNet34 [36] and DenseNet101 [37] as backbone). The comparison focused on metrics such as error, speed, model size, and computation time. To better simulate the noise encountered in real X-ray images, we conducted a robustness test to evaluate the adaptability of different methods under varying noise levels. Specifically, Poisson distribution was employed to model the photon noise generated during the random emission, propagation, and detection of X-ray photons. Moreover, Gaussian distribution is utilized to simulate random signal fluctuations (i.e., thermal noise and shot noise) that arise from the detector and its readout electronic system during X-ray imaging [38]. Three levels (low, medium, and high) of Poisson–Gaussian noise were added to the test database, as shown in Figure 6, simulating the effects of quantum noise and sensor electronic noise. The noisy X-ray images were then used as inputs to assess the registration accuracy of each method mentioned above, allowing for a comparison of their robustness under different noise conditions. Additionally, to verify the rationale and necessity of each component in our proposed method, we conducted an ablation analysis.

To ensure a fair comparison, all comparative experiments were performed under the same conditions, where the training and validation datasets, hyperparameters, and test hardware configurations remained consistent.

## 4. Results and Discussion

### 4.1. Evaluation Matrix

The mean absolute errors (MAE) between regression pose and ground truth pose on [α, β, θ, dx, dy, dz], mean target registration error (mTRE), and gross failure rate (GFR) were selected as the evaluation matrix, following the common evaluation indicators for 2D–3D registration described in Van et al.’s work [39], and mTRE is defined in Equation (11).

(11) $$mTRE=\frac{1}{n}\;\sum_{i=1}^{n}{\left\|T_{reg}\cdot P-T_{gt}\cdot P\right\|}_{2}\;,$$

where $T_{reg}$ and $T_{gt}$ represents the regressed and ground truth rigid transform matrix and P represents an anatomical 3D landmark point in the patient. mTRE calculates the mean distance (mm) between the ground truth pose and the estimate pose, which is applied to measure the accuracy of registration. Registration failure is usually defined as the conditions where mTRE>10 mm [40] in medical practice. GFR is defined as the proportion of registration failures among all samples.

In order to show the registration accuracy and uncertainty in different situations, MAE and mTRE were presented in the form of mean value ± standard deviation.

### 4.2. Comparison with Traditional Optimization-Based and DL-Based X-Ray 2D–3D Registration Algorithms

**Figure 7:**
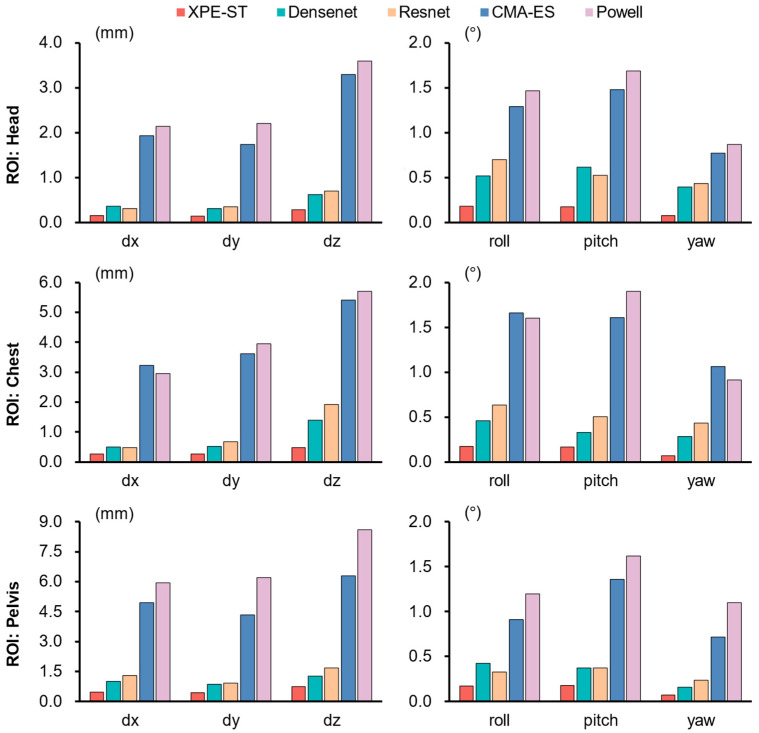
Mean absolute error of the six-degrees-of-freedom (6-DoF) registration parameters across different regions of interest (ROIs).

Both optimization-based and learning-based methods were utilized to benchmark registration performance against our proposed XPE-ST method. Among the optimization-based methods, we selected Powell [8] and CMA-ES [10], two classical nonlinear optimization approach commonly used in registration problems, as a representative method for comparison. The initial pose was randomly generated within the ranges of [−10°, 10°] for rotations and [−20 mm, 20 mm] for translations around the ground truth. GradNCC [41] loss based on the intensity of the whole image served as objective function to measure the similarity between the optimized guess and the target image. To control the regression time, the termination condition was set to a GradNCC difference of less than 10^−3^ or after 100 optimization iterations. Among the deep learning-based methods, we selected two widely used network architectures for parameter regression, single-channel ResNet34 [36], and DenseNet101 [37], as backbone structure and trained them on the same dataset. Figure 7 illustrates the accuracy comparison of each method in registering the six-degrees-of-freedom (6-DOF) posture parameters. Table 3 presents a comprehensive comparison of the mean translation and rotation errors, along with their associated uncertainties, across various registration methods. To facilitate a more direct and intuitive comparison, Figure 7 illustrates the mean errors for each individual degree of freedom.

The results show that learning-based registration methods achieve higher registration accuracy for each degree of freedom compared to traditional optimization-based methods. Notably, the optimization-based methods demonstrate sensitivity to the region of interest, exhibiting higher registration errors due to their tendency to fall into local optima when similar patterns are encountered. Furthermore, the experiment was designed with an extensive capture range, increasing the likelihood of optimization-based methods becoming entrapped in wrong exploration trajectories, which leads to greater uncertainty in the accuracy. Moreover, uneven grayscale distributions across different regions of interest can further impact the registration performance. Among the learning-based methods, XPE-ST outperforms others in regression accuracy across all degrees of freedom. Within each region of interest, the registration accuracy of XPE-ST is approximately 10 times that from the conventional optimization-based methods, and 2 times that from the learning-based techniques. This superior performance is attributed to the Swin transformer backbone’s robust feature extraction capabilities and the task-specific design of each module, which enhances the model’s overall precision in posture registration. It is worth noting that translation in the z-direction (dz) is more challenging to accurately estimate than other poses. This difficulty arises because small movements along the X-ray source to detector axis cause distortions in the anterior–posterior (AP) X-ray images that are hard to capture, which further increases the complexity of registration.

**Figure 8:**
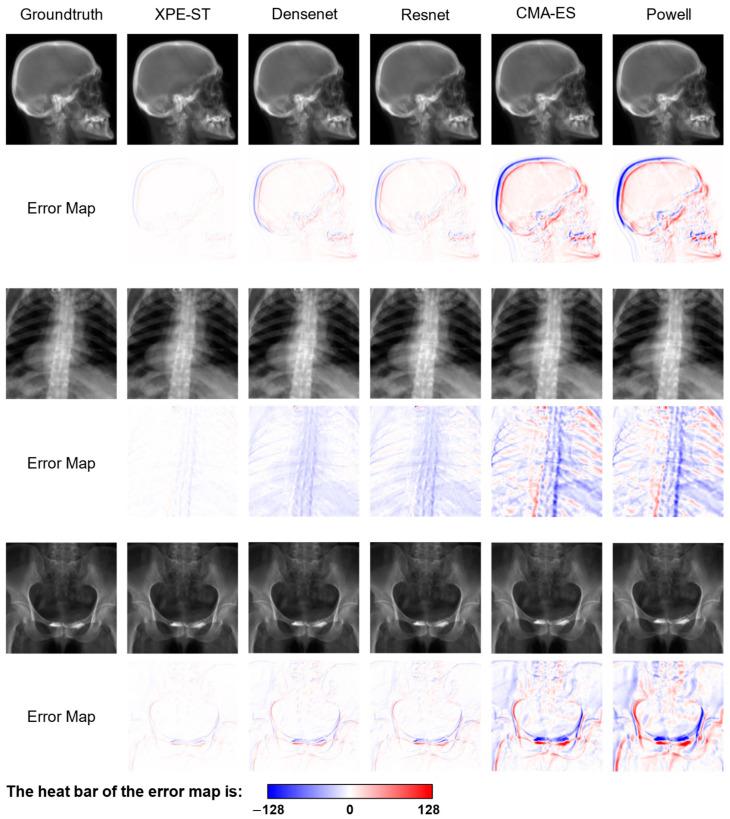
Visualization of the 2D–3D registration errors across different methods.

Additionally, Figure 8 visualizes the error between the ground truth image and the predicted image, obtained by applying the registered pose, to provide a more intuitive display of regression error differences. In the error map, regions appearing white indicate minimal grayscale differences between the two images, whereas areas close to red (or blue) signify substantial positive (or negative) grayscale discrepancies. The visualization directly demonstrates that XPE-ST offers superior registration accuracy, as evidenced by the predominance of white regions. Additionally, the grayscale errors exhibit a scaling pattern, corroborating the earlier observation that the challenge of estimating dz constitutes the primary source of error.

Table 4 summarizes the comparison of other registration indicators. The difference in mTRE remains consistent with regression accuracy across methods. Learning-based approaches show no failure cases in any region of interest, whereas optimization-based methods exhibit varying failure rates depending on the region. The failure of optimization methods can be attributed to not only the distinct grayscale distributions but also variations in the capture range. For example, in the pelvic region, the larger capture range in translation parameters expands the optimization searching space, which introduces more local optima, making it more challenging for the objective function to converge globally.

In terms of regression time, the optimization-based method requires the repeated generation of DRR images at various angles during its nonlinear exploration, which is computationally intensive and affected by DRR generation speed. For instance, head CT volumes are typically larger than other regions, requiring ray casting through more voxels, which prolongs DRR generation for a given number of iterations and thus results in the longest processing time. Conversely, the learning-based method requires only a single loading of the pre-trained model during prediction, with speed primarily dependent on the model size and system I/O. Consequently, all learning-based methods operate at comparable speeds, far outperforming traditional methods and meeting real-time registration requirements.

In terms of model size, ResNet has a smaller training footprint due to its skip connection characteristic, which reduces parameter counts compared to the dense connections in DenseNet. XPE-ST, while having a relatively larger training volume per single channel due to its additional modules, mitigates training resource demands by caching the precomputed feature map of the reference image. This caching allows for single-channel training during subsequent training iterations, effectively lowering resource consumption.

### 4.3. Noise Robustness Testing

**Figure 9:**
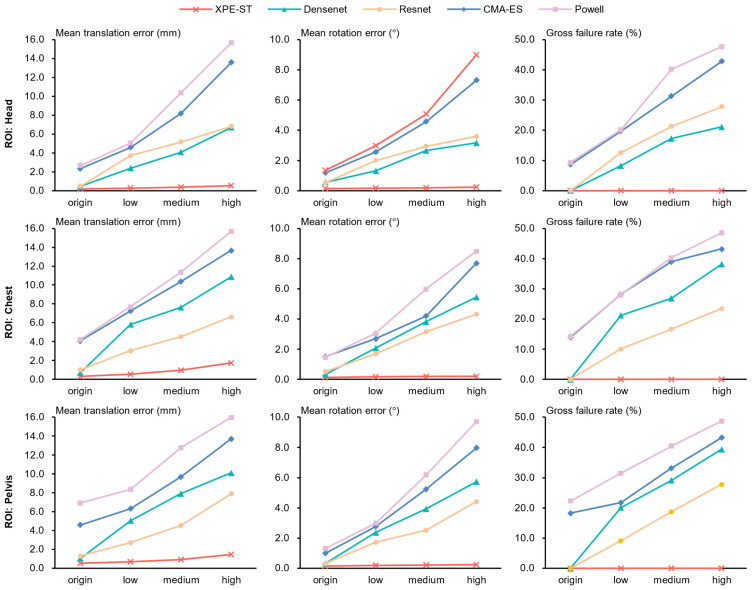
Comparison of the robustness of different methods across different noise levels in three regions of interest (ROIs).

We evaluated the robustness of the various methods outlined in Section 4.2 under different noise levels, with the results displayed in Table 5 and Figure 9.

The figure clearly illustrates that the traditional optimization method has the poorest adaptability to noise, with a registration failure rate approaching 50% under high noise levels. This vulnerability arises because added noise substantially alters the grayscale distribution of the images, making the objective function more non-convex and thus disrupting the converge accuracy. In comparison, ResNet and DenseNet exhibit some adaptability to noise; however, this robustness diminishes significantly under high noise conditions, resulting in a registration failure rate of approximately 30%. Even DenseNet, which showed superior accuracy than ResNet in previous evaluations, demonstrates lower noise adaptability and shows high error uncertainties. This sensitivity arises because these networks learn a mapping between detailed image features and parameters. When noise is introduced, the image features are substantially disrupted, impacting DenseNet more heavily due to its heightened sensitivity to fine-grained features.

Our proposed method, XPE-ST, exhibits minimal sensitivity to noise in translation parameters, and rotation parameters remain virtually unaffected, making XPE-ST the only method that consistently maintains a 0% failure rate across all noise levels. This adaptivity is attributed to the excellent performance of our Swin transformer backbone, which effectively captures both local and global multi-level information while leveraging a spatial attention mechanism to isolate the most valuable features. Furthermore, the squeeze-and-excitation channel attention and cross-stage feature pyramid maximize feature consistency across different levels, enhancing the system’s overall robustness and stability against noise.

### 4.4. Ablation Study

To comprehensively analyze the impact of each proposed module in XPE-ST, we tested the following four system configurations: single channel Swin transformer without and specific design, the dual-channel backbone only (DC), the dual-channel backbone with squeeze-and-excitation channel attention module (DC + SE) for feature reweighting, and the entire system including feature pyramid network (DC + SE + FPN) for feature fusion. The mean translation and rotation error and their corresponding uncertainties between the predicted pose and the ground truth pose using different configurations in three regions of interest is shown in Table 6.

It can be seen from Table 4 that the addition of each module contributes positively to reducing regression error and is indispensable for optimal performance. Among these modules, the dual-channel design has the most significant impact on improving accuracy, as it transforms the original mapping from images to poses into a mapping from image differences to poses, which can effectively highlight variations relevant to pose adjustments, thereby enhancing the model’s sensitivity to subtle changes. The SE and FPN modules enhance model accuracy to a similar degree by effectively reweighting features and fusing multi-level image information, which strengthens the model’s ability to capture detailed patterns and improves its generalization capabilities.

## 5. Conclusions

In this paper, we proposed an innovative method, X-ray pose estimation Swin transformer (XPE-ST), for preoperative CT and intraoperative X-ray registration. Preoperative CT was used to create digitally reconstructed radiography (DRR), i.e., a simulated X-ray image for further input. A dual-channel feature extraction Swin transformer equipped with a multi-level feature reweighting and fusion module was trained to predict the pose in six-degrees-of-freedom, i.e., x, y, z, pitch, yaw, and roll. To evaluate the performance of our proposed method, we collected open-source head, chest, and pelvis CT datasets, which were clinically relevant regions of interest, for comparative experiments. The experimental results showed that our method can obtain high robustness and pose estimation accuracy (mean rotation and translation error of 0.142° and 0.362 mm) in an extremely short time (0.02 s), which improves the registration speed and accuracy by about 1000 times and 15 times, respectively, compared with traditional optimization algorithms. We applied noise of varying intensities to simulate the differences between real and simulated X-rays, testing our model’s robustness under these conditions. The results demonstrate that XPE-ST is the only method capable of maintaining zero registration failures across different noise levels. The advantages in speed, accuracy, and robustness highlight the significant potential for future applications in surgical navigation, targeted radiotherapy, and other clinical procedures. Future work will focus on incorporating more real X-rays, leveraging style transfer for domain adaptation, and utilizing multi-view X-rays to expand the applicability prospects of our proposed method across a broader range of clinical scenarios. In addition, we will also combine medical image segmentation with deformation field simulation to match the 2D and 3D patterns in non-rigid regions.

## Figures and Tables

**Table 1:** Geometric parameters of the CT scans selected among different regions of interest.

Region of Interest	CT Pixel Resolution (L×W×H) ^1^	Voxel Spacing (mm)	Voxel Origin Offset (a,b,c) ^1^
Head	1024 × 1024 × 202	0.558 × 0.558 × 2	(−286, −208, −759)
Chest	512 × 512 × 176	0.694 × 0.694 × 2	(−168, 140, −1029)
Pelvis	512 × 512 × 350	0.740 × 0.740 × 0.8	(−168, −189, −1411)

^1^*L*, *W*, *H*, *a*, *b* and *c* are parameters illustrated in Equation (5).

**Table 2:** Capture range of 6-DOF registration pose parameters and number of training images among different regions of interest.

Parameters ^1^	Region of Interest		
	Head	Chest	Pelvis
dx range (mm)	[−105, −75] ^1^	[−25, 25]	[−25, 25]
dy range (mm)	[−15, 15]	[−15, 15]	[−25, 25]
dz range (mm)	[30, 90] ^1^	[−30, 30]	[−50, 50]
roll range (°)	[−15, 15]	[−15, 15]	[−105, −75] ^2^
pitch range (°)	[−15, 15]	[−15, 15]	[−15, 15]
yaw range (°)	[−5, 5]	[−5, 5]	[−5, 5]
Number of training images	500,000	750,000	1,000,000

^1^ Since the original CT scan encompasses both the head and neck, some translation parameters need to be adjusted in advance to ensure that the region of interest is correctly centered on the head. ^2^ The original CT scan needs to be rotated to achieve the anterior–posterior (AP) view of the pelvis.

**Table 3:** Mean translation and rotation errors across different registration methods in three regions of interest (ROIs).

Regions of Interest	Method	Mean Translation Error (mm) ^1^	Mean Rotation Error (°) ^1^
Head	Powell	2.6 ± 3.1	1.3 ± 1.5
	CMA-ES	2.3 ± 2.6	1.2 ± 1.2
	Resnet	0.46 ± 0.50	0.51 ± 0.58
	Densenet	0.40 ± 0.44	0.55 ± 0.60
	XPE-ST	**0.19 ± 0.16 ^2^**	**0.15 ± 0.12**
Chest	Powell	4.2 ± 5.0	1.5 ± 1.8
	CMA-ES	4.1 ± 4.5	1.5 ± 1.6
	Resnet	1.0 ± 1.1	0.52 ± 0.54
	Densenet	0.77 ± 0.86	0.35 ± 0.40
	XPE-ST	**0.34 ± 0.31**	**0.14 ± 0.13**
Pelvis	Powell	6.9 ± 8.1	1.3 ± 1.7
	CMA-ES	5.0 ± 5.3	0.99 ± 1.14
	Resnet	1.3 ± 1.5	0.31 ± 0.38
	Densenet	1.0 ± 1.3	0.32 ± 0.35
	XPE-ST	**0.56 ± 0.49**	**0.14 ± 0.12**

^1^ The mean absolute errors for d_x_, d_y_, and d_z_ (roll, pitch, and yaw) are further averaged to yield an overall mean translation (rotation) error metric. ^2^ The bolded content highlights the most outstanding metric in the comparison.

**Table 4:** Comparison of different registration methods in mean target registration error (mTRE), gross failure rate (GFR), regression time, and memory usage (during training) across different regions of interest (ROIs).

Regions of Interest	Method	mTRE (mm)	GFR	Time (s)	Memory Usage (Gb) ^1^
Head	Powell	4.3 ± 5.6	9.31%	43.62	/
	CMA-ES	4.0 ± 4.9	8.63%	46.87	/
	Resnet	0.79 ± 1.0	**0.00% ^2^**	**0.0067**	**12.98**
	Densenet	0.71 ± 0.75	**0.00%**	0.022	36.62
	XPE-ST	**0.32 ± 0.28**	**0.00%**	0.015	28.04
Chest	Powell	7.3 ± 7.1	14.23%	21.04	/
	CMA-ES	7.1 ± 6.9	13.89%	23.29	/
	Resnet	1.9 ± 2.2	**0.00%**	**0.0067**	**12.98**
	Densenet	1.0 ± 1.3	**0.00%**	0.022	36.62
	XPE-ST	**0.41 ± 0.39**	**0.00%**	0.015	28.04
Pelvis	Powell	12 ± 10	22.30%	29.75	/
	CMA-ES	8.0 ± 7.8	18.26%	31.56	/
	Resnet	2.0 ± 2.2	0.00%	**0.0067**	**12.98**
	Densenet	1.6 ± 2.0	0.00%	0.022	36.62
	XPE-ST	**0.79 ± 0.73**	**0.00%**	0.015	28.04

^1^ Memory usage within single channel, batch size is set to 128. ^2^ The bolded content highlights the most outstanding metric in the comparison.

**Table 5:** Mean translation and rotation errors under different noise levels across three regions of interest (ROIs).

Regions of Interest	Method	Mean Translation Error (mm)			Mean Rotation Error (°)		
		Low	Medium	High	Low	Medium	High
Head	Powell	5.0 ± 6.2	10 ± 10	16 ± 14	3.0 ± 3.4	5.1 ± 5.0	9.0 ± 7.8
	CMA-ES	4.6 ± 5.2	8.2 ± 9.1	14 ± 15	2.6 ± 3.1	4.6 ± 4.2	7.3 ± 7.7
	Resnet	3.7 ± 4.5	5.2 ± 6.5	6.8 ± 8.0	2.0 ± 2.3	2.9 ± 3.3	3.6 ± 4.3
	Densenet	2.4 ± 3.0	4.1 ± 5.2	6.7 ± 7.9	1.3 ± 2.1	2.7 ± 3.8	3.2 ± 4.5
	XPE-ST	**0.24 ± 0.20 1**	**0.36 ± 0.45**	**0.54 ± 0.88**	**0.16 ± 0.15**	**0.18 ± 0.28**	**0.24 ± 0.39**
Chest	Powell	7.7 ± 8.9	11 ± 13	16 ± 15	3.1 ± 4.2	6.0 ± 5.4	8.5 ± 8.2
	CMA-ES	7.3 ± 8.2	10 ± 12	14 ± 14	2.7 ± 3.5	4.2 ± 4.8	7.7 ± 8.1
	Resnet	3.1 ± 4.1	4.5 ± 5.8	6.6 ± 7.9	1.7 ± 2.3	3.2 ± 5.0	4.3 ± 6.2
	Densenet	5.8 ± 6.4	7.6 ± 9.2	10.9 ± 12.0	2.1 ± 2.8	3.8 ± 4.5	5.5 ± 7.3
	XPE-ST	**0.56 ± 0.64**	**0.98 ± 1.11**	**1.7 ± 2.2**	**0.17 ± 0.20**	**0.19 ± 0.23**	**0.21 ± 0.34**
Pelvis	Powell	8.4 ± 8.9	13 ± 14	16 ± 15	3.0 ± 3.3	6.2 ± 5.3	9.7 ± 8.7
	CMA-ES	6.3 ± 7.5	10 ± 10	14 ± 12	2.8 ± 2.6	5.2 ± 6.0	8.0 ± 7.1
	Resnet	2.7 ± 3.4	4.5 ± 5.9	7.9 ± 9.1	1.7 ± 2.4	2.5 ± 3.9	4.4 ± 5.8
	Densenet	5.1 ± 6.7	7.9 ± 9.5	10 ± 12	2.4 ± 2.9	3.9 ± 4.8	5.7 ± 7.0
	XPE-ST	**0.68 ± 0.89**	**0.91 ± 1.66**	**1.5 ± 2.0**	**0.19 ± 0.26**	**0.21 ± 0.31**	**0.24 ± 0.44**

^1^ The bolded content highlights the most outstanding metric in the comparison.

**Table 6:** Comparison of different combinations in XPE-ST (dual channel design ‘DC’, squeeze-and-excitation channel attention ‘SE’, and feature pyramid network ‘FPN’) for pose registration performance. In the table, ‘**×**’ indicates that the module is not enabled, while ‘**√**’ indicates that the module is enabled.

Regions of Interest	DC	SE	FPN	Mean Translation Error (mm) ^1^	Mean Rotation Error (°) ^1^
Head	**×**	**×**	**×**	0.48 ± 0.60	0.55 ± 0.69
	**√**	**×**	**×**	0.30 ± 0.30	0.31 ± 0.36
	**√**	**√**	**×**	0.23 ± 0.20	0.24 ± 0.22
	**√**	**√**	**√**	**0.19 ± 0.16 ^2^**	**0.15 ± 0.12**
Chest	**×**	**×**	**×**	1.13 ± 1.30	0.52 ± 0.62
	**√**	**×**	**×**	0.60 ± 0.71	0.29 ± 0.34
	**√**	**√**	**×**	0.45 ± 0.48	0.21 ± 0.22
	**√**	**√**	**√**	**0.34 ± 0.31**	**0.14 ± 0.13**
Pelvis	**×**	**×**	**×**	1.29 ± 1.38	0.51 ± 0.60
	**√**	**×**	**×**	0.70 ± 0.87	0.32 ± 0.39
	**√**	**√**	**×**	0.62 ± 0.62	0.24 ± 0.26
	**√**	**√**	**√**	**0.56 ± 0.49**	**0.14 ± 0.12**

^1^ The mean absolute errors for dx, dy, and dz (roll, pitch, and yaw) are further averaged to yield an overall mean translation (rotation) error metric. ^2^ The bolded content highlights the most outstanding metric in the comparison.

## Data Availability

The authors confirm that all the medical image databases used for supporting the findings of this study are open-access and are available within the article.
